# Complete mitochondrial genome of the river cooter (*Pseudemys concinna*, Testudines: Emydidae) in Korea

**DOI:** 10.1080/23802359.2022.2119820

**Published:** 2022-09-15

**Authors:** Jaehong Park, Seungju Cheon, Seung-Min Park, Ha-Cheol Sung, Dong-Hyun Lee

**Affiliations:** a School of Biological Sciences and Biotechnology Graduate School, Chonnam National University, Gwangju, South Korea; b Research Center of Ecomimetics, Chonnam National University, Gwangju, South Korea; c Department of Biological Sciences, College of Natural Sciences, Chonnam National University, Gwangju, South Korea

**Keywords:** *Pseudemys concinna*, Emydidae, mitochondrial genome

## Abstract

The complete mitochondrial genome of *Pseudemys concinna* in Korea was sequenced and characterized. The mitochondrial genome is constituted of 37 genes (13 protein-coding genes, 22 transfer RNA (tRNA) genes, and two ribosomal RNA (rRNA) genes) and a noncoding region. Phylogenetic analysis based on the complete mitochondrial genome showed that *P. concinna* has closer relationship with *Chrysemys picta* than *Trachemys scripta elegans.* This is the first case for complete mitochondrial genome from *P. concinna* in Korea, which will provide information for biogeographical studies and management plan for invasive species.

The river cooter (*Pseudemys concinna*, Le Conte 1830) is native to the central and eastern United States (Ward and Jackson 2008). However, as import for pet trade and intentional release have increased, the river cooter has spread widely in other countries including Korea (Kim et al. 2020; Koo et al. 2020). In 2020, the Ministry of Environment of Korea classified the river cooter as an invasive species (Kim et al. 2020). The invasive species can compete with the native species, leading to a decrease in the number of the native species (Hayes et al. 2018; Koo et al. 2020). Also, it is possible that the invasive species disturb the genetic background of native species (Parham et al. 2013; Meilink et al. 2015). Despite the potential risk, the survey for the invasive species is still insufficient. In addition, the complete mitochondrial genome of the river cooter has not been identified, though only a portion of its genetic information has been known (Spinks et al. 2013). In this study, we sequenced the complete mitochondrial genome of *Pseudemys concinna*, and these data can help phylogenetic studies and the management of the invasive species.

The *P. concinna* specimen was collected from Gwangju (35°10′29.25″N, 126°54′36.46″E), Korea, and the total genomic DNA was extracted from the tail using the DNeasy Blood & Tissue kit (Qiagen, Valencia, CA) according to the manufacturer’s protocol. The extracted DNA sample was deposited at the Museum of Wildlife, located in Research Center of Ecomimetics, Chonnam National University, South Korea (specimen accession number: 2021-RCE-PC001; shcol2002@chonnam.ac.kr). The mitochondrial genome was analyzed using Illumina HiSeqXten platform (Illumina, San Diego, CA), which was performed by Macrogen (Seoul, South Korea). Raw sequence data were checked by FastQC, and adaptor trimming and quality filtering were performed by Trimmomatic (Andrews 2010; Bolger et al. 2014). Subsequently, *de novo* assembly was conducted using SPAdes and the filtered reads were aligned using BLAST (Altschul et al. 1990; Bankevich et al. 2012). Finally, the complete sequence was annotated using MITOS2 web server (Bernt et al. 2013).

The complete mitochondrial genome of *P. concinna* is 16,738 bp in length deposited in GenBank (accession number: OM935747), and contains 13 protein-coding genes, 22 transfer RNA (tRNA) genes, two ribosomal RNA (rRNA) genes, and a putative long non-coding control region. Twelve protein-coding genes, 14 tRNA genes, and two rRNA genes are encoded in heavy strand, whereas one protein-coding gene (NADH dehydrogenase subunit 6) and eight tRNA genes in light strand. The nucleotide composition of the *P. concinna* mitochondrial genome (A = 34.5%, T = 26.6%, C = 25.9%, and G = 12.9%) is similar to that of *T. scripta elegans* Korea (MW019443; A = 34.3%, T = 27.0%, C = 25.9%, and G = 12.9%), *Mauremys sinensis* China (KC333650; A = 33.9%, T = 26.3%, C = 26.6%, and G = 13.2%), *Mauremys reevesii* Korea (KJ700438; A = 34.1%, T = 27.0%, C = 26.1%, and G = 12.8%), and *Chrysemys picta* USA (AF069423; A = 34.4%, T = 26.8%, C = 25.9%, and G = 12.8%). The sequence of *P. concinna* has higher similarity with that of *C. picta* (93%) than other turtles including *T. scripta elegans* (90%), *M. sinensis* (81%), and *M. reevesii* (80%).

To investigate the phylogenetic position of *P. concinna*, the complete mitochondrial genome sequences of 14 species in the order Testudines were extracted from GenBank. To check the possibility of genetic disturbance by invasive species in Korean native species, we chose *M. reevesii* and *P. sinensis* as a native species and *M. sinensis*, *M. temminckii*, *C. picta*, and *T. scripta* as an invasive species (Song et al. 2012; Koo et al. 2020). Also, we took data analyzed in Korea and other countries including China, U.S.A., and Canada to compare the difference between countries. The phylogenetic tree was constructed using MEGA X software (Figure 1; Kumar et al. 2018). Specifically, the sequences were aligned using MUSCLE algorithm and the phylogenetic tree was made using maximum-likelihood method and Tamura–Nei model with 1000 bootstrap replicates (Tamura and Nei 1993; Edgar 2004). In agreement with sequence identity data, *P. concinna* is closer with *C. picta* than *T. scripta elegans*. But *P. concinna* is separated from *C. picta* in the phylogenetic tree. These data provide information on the complete mitochondrial genome of *P. concinna* for the first time and can contribute to further studies on biodiversity and management of *P. concinna* which is an invasive species in many countries including Korea.

**Figure 1.**
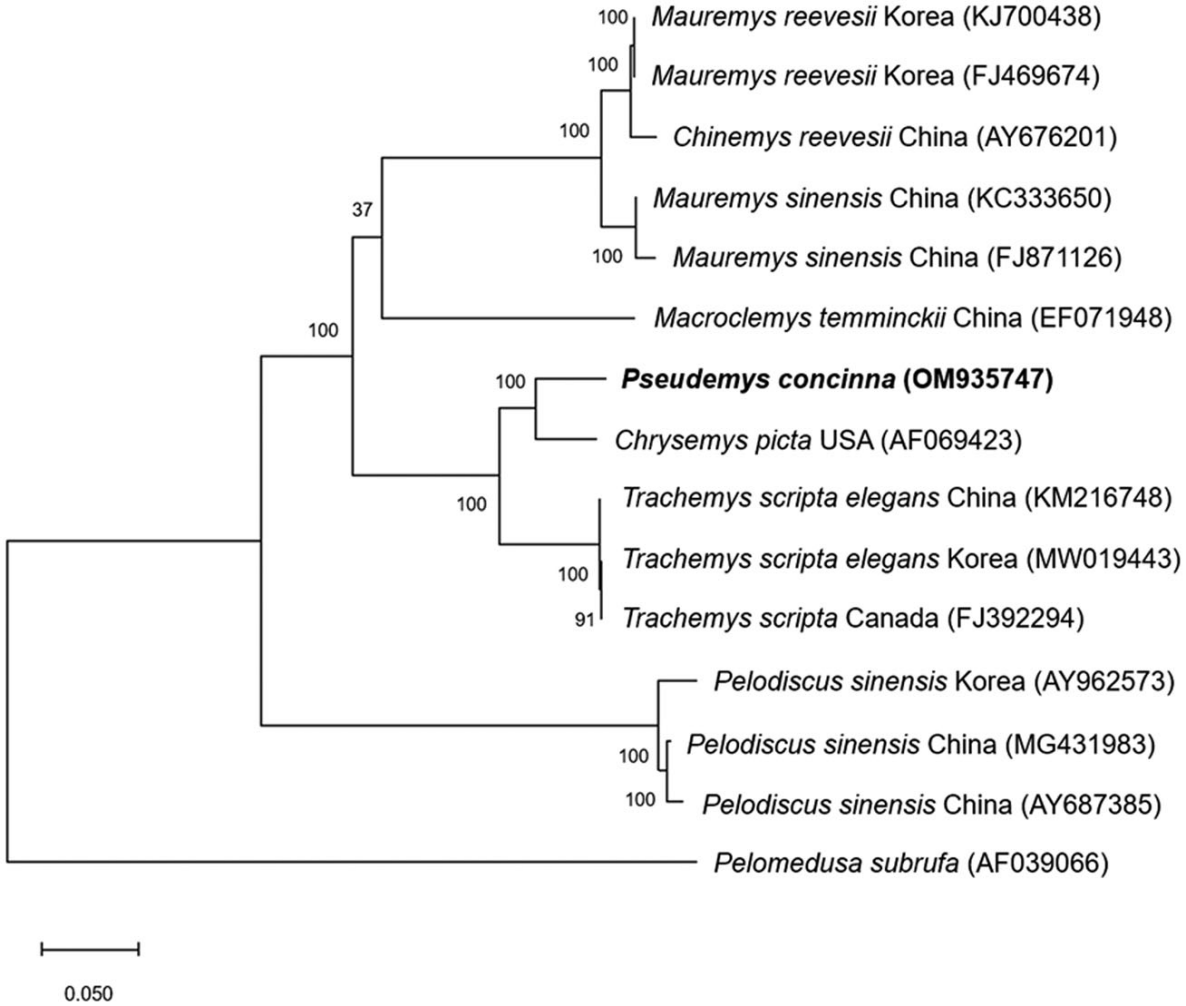
Phylogenetic tree of *Pseudemys concinna* and other related species based on complete mt genome sequences. Phylogenetic analysis was performed using MEGA X software. GenBank accession numbers of each mt genome sequence are given in the bracket after the species name, and the bootstrap value based on 1000 replicates is represented on each node. *Pelomedusa subrufa* was used as outgroup to root the tree.

## Data Availability

GenBank accession number from the complete mitochondrial genome of *Pseudemys concinna* (OM935747) has been registered with the NCBI database (https://www.ncbi.nlm.nih.gov/OM935747). The associated BioProject, BioSample, and SRA accession numbers are PRJNA813976, SAMN26520676, and SRR18578428, respectively.
